# Poor Yield of Routine Transthyretin Screening in Patients with Idiopathic Neuropathy

**DOI:** 10.1017/cjn.2020.114

**Published:** 2020-11

**Authors:** Dina Namiranian, Colin Chalk, Rami Massie

**Affiliations:** Department of Neurology and Neurosurgery, Faculty of Medicine, McGill University, Montreal, QC, Canada

**Keywords:** Transthyretin, Idiopathic polyneuropathy, Genetic screening, Amyloid

## Abstract

**Background and objectives:**

Transthyretin familial amyloid polyneuropathy (TTR-FAP) is caused by a mutation in the transthyretin (TTR) gene. Although classically described as rapidly progressive and life-threatening, recent studies on TTR-FAP show significant genetic and phenotypic heterogeneity depending on geographic localization. In light of new therapeutic advances and their implication for patient management, the aim of our study was to determine the prevalence of TTR-FAP within patients with idiopathic neuropathy in a North American population.

**Methods:**

We sequenced the TTR gene in a cohort of patients with idiopathic neuropathy. Genetic screening was performed in 110 patients from two neuromuscular clinics in Montreal, Canada.

**Results:**

No variants of unknown significance or pathogenic mutations were detected in the TTR gene.

**Conclusion:**

Our study confirms that TTR-FAP is a rare entity in our patient population, and that diagnostic yield of screening all patients with idiopathic neuropathy is very low.

## RÉSUMÉ

**Efficacité négligeable d’un dépistage de routine de la transthyrétine chez des patients atteints de neuropathie idiopathique**.

**Contexte et objectifs:**

La polyneuropathie amyloïde héréditaire à transthyrétine est causée par la mutation du gène de la transthyrétine (TTR). Bien que généralement décrite comme une maladie génétique mortelle qui évolue rapidement, des études récentes ont suggéré que la polyneuropathie amyloïde héréditaire à transthyrétine donne à voir une grande hétérogénéité génétique et phénotypique selon l’emplacement géographique des patients. À la lumière des nouvelles avancées thérapeutiques et de leurs implications dans la prise en charge des patients, le but de notre étude est de déterminer la prévalence de cette maladie génétique chez des patients nord-américains atteints de neuropathie idiopathique.

**Méthodes:**

Nous avons séquencé le gène TTR au sein d’une cohorte de patients atteints de neuropathie idiopathique. Un dépistage génétique a été ensuite mené chez 110 patients ayant été suivis dans deux cliniques neuromusculaires de Montréal (Canada).

**Résultats:**

Aucune variante génétique d’importance inconnue ou de mutation pathogène n’a été détectée dans le gène TTR.

**Conclusion:**

Notre étude confirme donc que la polyneuropathie amyloïde héréditaire à transthyrétine est un phénomène peu fréquent au sein de notre groupe de patients. En cela, l’efficacité diagnostique d’un dépistage de tous les patients atteints de neuropathie idiopathique demeure faible.

## Introduction

Transthyretin (TTR) is a 127-amino-acid polypeptide mainly synthesized in the liver, which assembles to form a tetrameric protein that circulates in the bloodstream to transport thyroxin (T4) and retinol. Mutations in the TTR gene destabilize the TTR tetramers and favor their dissociation into monomers that self-aggregate to form non-fibrillar soluble oligomers and protofibrils. These in turn assemble into insoluble amyloid fibrils which deposit in multiple organs, predominantly the heart and peripheral nerves.^1^

Transthyretin familial amyloid polyneuropathy (TTR-FAP) is an autosomal dominant disease caused by mutations in the TTR gene. At present, 119 point mutations in the TTR gene have been identified, the most common being Val30Met in Portuguese, Swedish, and Japanese populations.^2^ Typically, patients present in their mid-20–40s with a rapidly progressing sensorimotor, length-dependent polyneuropathy, which predominantly affects unmyelinated and small myelinated fibers. Nerve conduction studies classically demonstrate an axonal pattern of injury; however, the possibility of nerve conduction studies in the demyelinating range has been described in late onset TTR-FAP.^4–6^ Other common symptoms involve autonomic disturbance and extra-neurological manifestations such as cardiomyopathy, vitreous involvement, and progressive renal failure.^1^ Death within 10 years is believed to be the usual outcome.^3^

In the past, management of this disorder was restricted to symptomatic treatment of neuropathic symptoms and liver transplantation, although the latter was often undertaken relatively late in the disease course because of delays in diagnosis. Liver transplantation improves quality of life but does not often reverse or alter the progression of organ impairment.^7^ In recent years, new therapeutic options using TTR stabilization molecules (diflunisal and tafamidis) have been found to be efficacious in slowing down the progression of the neuropathy compared to placebo.^8,9^ Furthermore, in July 2018, two gene-silencing therapies (patisiran and inotersen) proved to be successful in two phase III trials, opening a promising new avenue in treating this disease.^10,11^ These new treatments are reported to slow down and occasionally reverse the progression of the neuropathy in patients with TTR-FAP Stage 1 or 2.^12^ Correctly identifying TTR-FAP, and differentiating it from acquired forms of amyloid neuropathy that are treated with potentially harmful chemotherapy agents, carries important therapeutic implications.

Given the severity of the natural course of the disease and the new possibilities for therapeutics, there is increasing emphasis on the importance of adequately recognizing and testing for TTR-FAP. Nerve or muscle biopsies are often unreliable due to random deposition of amyloid. With genetic testing becoming widely available, it has been observed that clinical phenotypes of TTR-FAP are more diverse than originally described, and that there is strong genetic heterogeneity depending on geographic localization.^13,14^ In addition, we and others have wondered if TTR-FAP is more prevalent than previously thought, particularly as a considerable proportion of patients present as isolated cases, without a family history.^4^ Although many studies have characterized prevalence and genotypes in Europe and Asia, relatively little is known about the prevalence and phenotypes of TTR-FAP in North America.^14^ Hence, the current study was designed to identify the prevalence of TTR-FAP in patients labeled with a diagnosis of idiopathic neuropathy in a Canadian neuromuscular center.

## Material and Methods

We conducted a prospective study in two specialized neuromuscular clinics within the McGill University Health Center (MUHC) in Montreal, Canada. Genetic screening was performed between June and August 2019. All patients signed an informed consent form for the study approved by our local research ethics board (MUHC-REB). Patients were identified by searching the databases of the Montreal General Hospital neuropathy clinic (1014 patients seen since 2000) and the Montreal Neurological Hospital neuromuscular clinic (921 patients since 2010).

Patients included were adults previously diagnosed with a neuropathy, either clinically or electrophysiologically, for whom a clear underlying etiology was not identified by the treating neurologist. Patients had basic laboratory screening, which included HbA1c, thyroid-stimulating hormone (TSH), serum protein electrophoresis (SPEP), and vitamin B12. Alcoholic and toxic causes were screened by history. A workup for markers of connective tissue and systemic inflammatory disorders as well as infectious causes was performed on selected patients depending on their clinical presentation. Patients with a suspected genetic etiology were eligible, if exact genetic diagnosis had not been reached. A proportion of these patients had undergone prior genetic screening via genetic panels (23/110); these were reviewed and patients whose panels included TTR gene testing were excluded from the study. Patients with axonal neuropathies and monoclonal gammopathy of unknown significance (MGUS) were included, given the uncertainty about whether or not the MGUS was causative. Patients with demyelinating neuropathies (defined as conduction velocity <70% of lower limit of normal or presence of conduction block or temporal dispersion) and MGUS were excluded regardless of their anti-MAG status, as it was felt that the MGUS was more likely causative. Patients with idiopathic demyelinating inherited neuropathies and those with atypical acquired demyelinating neuropathies were included, given the few reports of TTR-FAP presenting with demyelinating range electrophysiology.

Genetic sequencing and duplication/deletion study was performed in the TTR gene on patients’ blood samples by Invitae Corporation. This sequence analysis covers all 4 exons, as well as 10 base pairs of adjacent intronic sequence, and select noncoding variants.

## Results

In this study, 293 out of 1935 patients followed at our clinics met our inclusion criteria and were contacted for participation in the study; 110 patients agreed to participate and underwent venepuncture. Table 1 lists the patients’ main neuropathy characteristics. The majority of our patient population presented with an axonal sensorimotor polyneuropathy (80/110), followed by idiopathic small fiber neuropathy (20/110). Among those 2 groups, 10 patients had MGUS. Ten patients had demyelinating neuropathies. Twenty-three out of 97 patients with a presumed inherited neuropathy had had prior genetic testing that did not include TTR in their panel and were contacted for the study. The most frequent ethnic background was French Canadian (51/110), followed by English Canadian (25), Italian (6), Polish (3), and British (3). Twenty-one patients were from diverse backgrounds, and we were unable to determine the origins of one patient.

**Table 1:** Neuropathy characteristics

Type of neuropathy	Number of patients (%)
Axonal polyneuropathy^*^	
Pure small fiber	20 (18%)
Sensorimotor	80 (73%)
Demyelinating polyneuropathy	
Suspected inherited	8 (7%)
Suspected acquired (but atypical features as per treating physician)	2 (2%)
Total	110

* Including 10 patients with monoclonal gammopathy of unknown significance (4 small fiber and 6 sensorimotor).

We identified no variants of unknown significance or pathogenic mutations in the TTR gene in the 110 patients tested.

We also reviewed the presence of red flag symptoms or signs in the patients tested (Table 2). About one third of patients tested (40/110) had one or more red flags, the most common being positive family history. There were very few patients with associated cardiac or renal disease of unclear etiology.

**Table 2:** TTR-FAP red flags within our tested population

Red flags	Number of patients affected (% of patients screened)
Any family history of neuropathy or high-arched feet	20 (18%)
Rapidly progressing polyneuropathy^a^	10 (9%)
Entrapment neuropathies	
Bilateral CTS	7 (6%)
Unilateral CTS	2 (2%)
Other	0
Associated cardiac or renal involvement	
Arrythmia	1 (1%)
Chronic kidney failure of unclear etiology^b^	1 (1%)
Cardiomyopathy of unclear etiology^b^	0
Autonomic symptoms	0

CTS = carpal tunnel syndrome.

a As determined clinically by the treating physician.

b Patients with ischemic cardiomyopathy or with clear alternate etiology for their cardiac or renal disease were not included.

## Discussion

This is the first study looking at the prevalence of TTR-FAP within a North American population with idiopathic neuropathy. Our finding that the prevalence of TTR-FAP in patients with idiopathic neuropathy is very low is in accordance with two recent studies. An Italian study^15^ screened 98 patients with an undiagnosed axonal sensorimotor polyneuropathy and found no pathogenic mutations in the TTR gene. A Scandinavian study^16^ screened 155 patients with a diagnosis of small fiber or mixed neuropathy without clear etiology and also found no pathogenic mutations in the TTR gene. The Scandinavian study mostly included patients with small fiber neuropathies, whereas our study was designed to be more inclusive in its selection criteria, given the phenotypic variability and the lack of clear phenotypic description of TTR-FAP in North America.

The results from our Canadian sample suggest that routine genetic screening for the TTR gene in all patients with neuropathy of unknown etiology is likely to be of low yield in North America. However, occasional undiagnosed cases of TTR-FAP are probably to be found in most large neuromuscular clinics, as demonstrated by a patient previously seen at our clinic described in Box 1.

Box 1. **Box 1:**

A 70-year-old man of Italian origin, known for coronary artery disease and obstructive sleep apnea, presented with a 30-year history of very slowly progressive purely sensory symptoms affecting his feet and more recently ascending to the knees. His family history was significant for his mother, who started having neuropathy symptoms at the age of 80 and became wheelchair bound within 5 years. He has three asymptomatic siblings, and one sibling who has had intermittent sensory symptoms for several years. The patient’s initial nerve conduction studies were normal, but a repeat study 7 years later showed a moderate axonal sensorimotor polyneuropathy. A presumptive diagnosis of hereditary sensory autonomic neuropathy (HSAN) type I was made initially, before he moved and was lost to follow-up. He consulted in another institution and was found to have a Val30met mutation in the TTR gene. His cardiac echography was normal. He was started on diflunisal and has had some improvement in his sensory symptoms and neuropathic pain.

The patient in Box 1 illustrated that TTR-FAP may have a much more indolent and benign course than is commonly expected, and that distinctive features such as dysautonomia or cardiomyopathy may be minimal or absent. Nevertheless, presence of red flags for TTR-FAP (positive family history, rapidly progressive neurological deficits, associated cardiac, renal, or ocular disease, autonomic dysfunction, inexplicable weight loss, and entrapment neuropathies—particularly bilateral carpal tunnel syndrome)^17^ should prompt consideration of TTR gene testing. Surprisingly, neuropathic pain seems not to be a common initial symptom of TTR-FAP, although it may be encountered more often in the late-onset Val30Met form of the disease.^17^

In the last few years, the TTR gene has been added to most neuropathy genetic test panels. Given their cost-effectiveness and practicality, genetic test panels are recommended over individual TTR gene testing. However, clinicians must ensure that the TTR gene is included in the panel requested if their patients have red flags for the condition.

In our clinic population, an additional 48 patients, including 4 patients with pure small fiber neuropathy, had already been screened for TTR gene mutations prior to our study, and had negative results. Four patients with TTR mutations have been seen in our clinics, each of them with a different mutation (patient #1, Val122lle mutation; patient #2, Asp38Ala mutation; patient #3, Val50Met mutation; and patient #4, Val30Met mutation). Each of these patients has at least one of the red flags listed above. Patients #1 and #2 presented with a cardiomyopathy without polyneuropathy and were found to have bilateral carpal tunnel syndrome. Patient #3, who had bilateral carpal tunnel syndrome, presented with an upper extremity predominant neuropathy, which appears to be a common presentation of TTR-FAP in non-endemic areas.^18^ Patient #4 is the patient described above. By comparison, most of the patients in our study did not have red flags for TTR-FAP, although 36% did, the most common being positive family history.

The main strength of our study is the inclusion of well-characterized patients with a wide range of neuropathy phenotypes, including demyelinating range neuropathies, likely similar to patients seen in other referral centers in North America. As almost half of our study patients were French Canadian, the generalizability of our findings to a more “typical” North American population is limited. However, the mixture of French Canadian, English Canadian as well as mixed backgrounds from Europe, the Middle East, North Africa, and the Caribbean is well representative of the Canadian population. Another limitation is the relatively small patient sample. It is conceivable that the substantial fraction of eligible patients who declined to participate (usually because of transportation difficulties) lead to a selection bias, but we think this is unlikely. Our findings do not allow us to estimate the prevalence of TTR-FAP among patients with idiopathic neuropathy, but they do indicate that the yield of routine TTR gene testing in all patients with idiopathic neuropathy is very low. Screening patients for red flags would be expected to increase the pre-test probability.

Although our study suggests that TTR-FAP remains a rare disorder, registries of patients with TTR mutations will improve understanding of the range of the disorder’s clinical spectrum and variability of its natural history. A Canadian Registry for Amyloidosis Research (CRAR) is currently being developed to better characterize the Canadian amyloidosis patient population and identify patients for clinical trials and novel therapies. The Transthyretin-Associated Amyloidosis Outcome Survey (THAOS) is a large observational international registry with a main goal of describing the natural course of TTR-FAP, as well as TTR-cardiomyopathy (both hereditary and wild-type).^19^ More recently, the publication of Canadian evidence-based guidelines for the evaluation and management of patients suspected to have cardiac amyloidosis should increase awareness of the disease and lead to better diagnosis.^20^ Along with the recent improved accessibility of genetic testing, better characterization of the variable phenotype of TTR-FAP will hopefully lead to better and earlier detection and eventually improved outcome in this disease.
